# Risk and protective factors for anastomotic insufficiency in elective colon and rectal cancer resections – a multivariate analysis with over 700 patients

**DOI:** 10.1007/s00423-025-03953-9

**Published:** 2026-01-09

**Authors:** Undine Gabriele Lange, Matthias Mehdorn, Gudula Keller, Anja Willing, Holger Nitzsche, Leif Christian Wagner, Ines Gockel, Boris Jansen-Winkeln, Soeren Torge Mees, Sigmar Stelzner

**Affiliations:** 1 Department of Visceral, Transplant, Thoracic and Vascular Surgery, University Hospital Leipzig, 04103 Leipzig, Germany; 2 Orthopedic Surgery, Caspar-David-Friedrich Str. 15, 01217 Dresden, Germany; 3 Department for General and Visceral Surgery, Dresden-Friedrichstadt General Hospital, 01067 Dresden, Germany; 4 Department for General, Visceral, Thoracic and Vascular Surgery, Federal Armed Forces Hospital Koblenz, 56072 Koblenz, Germany; 5 University Center for Gastrointestinal and Liver Disease, Clarunis, 4002 Basel, Switzerland; 6 Department for General, Visceral, Thoracic and Vascular Surgery, Hospital St. Georg Leipzig, 04129 Leipzig, Germany

**Keywords:** Colorectal cancer surgery, Risk factors, Bowel preparation, Oral antibiotics

## Abstract

**Purpose:**

This study aimed to identify the risk factors and protective factors for AL.

**Methods:**

Data from 717 patients with colorectal cancer with primary anastomosis who underwent elective surgery between 2012 and 2019 (418 patients with colon and 299 patients with rectal cancer) were extracted from prospectively established and retrospectively analyzed databases of two certified colorectal cancer centers, and processed for pre-, intra-, and postoperative risk factors in a multivariate analysis.

**Results:**

AL occurred in 33 (7.6%) patients with colon cancer and 30 (10%) patients with rectal cancer. AL was more likely after (extended) left hemicolectomy/sigmoidectomy (OR 3.771; 95% CI 1.17–12.154; *p* = 0.026) and in combination with perioperative administration of blood transfusions (OR 2.926; 95% CI 1.013–8.455; *p* = 0.047) in colon surgery. BMI ≥ 30 kg/m^2^ significantly increased the probability of AL (OR 7.136; 95% CI 2.248–12.651; *p* < 0.001) in rectal cancer surgery. Preoperative oral antibiotics, mainly in combination with mechanical bowel preparation, were associated with a reduced AL rate in both entities (OR 0.199, 95% CI 0.064–0.617; *p* = 0.005 for colon; OR 0.278, 95% CI 0.078–0.985; *p* = 0.047 for rectal cancer), regardless the antibiotic regimen used. The number required for treatment was calculated as 15.94.

**Conclusion:**

Our study, reflecting daily clinical practice, confirmed known risk factors (left-sided resection, perioperative blood transfusions, obesity) and identified oral antibiotics as the only protective factor against AL. The application of a single or double oral dose of two antibiotics is safe and pragmatic and should be implemented in standard operative procedures for colorectal cancer surgery.

**Supplementary Information:**

The online version contains supplementary material available at 10.1007/s00423-025-03953-9.

## Introduction

According to the current literature, anastomotic leakage (AL) following colorectal surgery occurs in 3–24% of patients [1–4] and represents a life-threatening condition that results in perioperative mortality of 2–24% with a 25% risk of permanent ostomy placement [5, 6]. In addition to the undisputed deterioration of short-term outcomes and significantly increased hospital costs [7], AL in the rectum is associated with increased rates of local recurrence and reduced overall and cancer-specific survival. With regard to colon cancer resection, there is no augmented risk of local recurrence; however, survival is diminished [8, 9].

The wide variance in the occurrence of AL is primarily due to the location of the anastomosis; less anastomotic leakage has been reported in right-sided hemicolectomy than in anastomosis of the left colon/rectum. Other risk factors for AL are the subjects of intensive research [10–12]. Previous studies have highlighted potential factors associated with outcomes are divided into preoperative (e.g., patient demographics, comorbidities, smoking, malnutrition, preoperative therapy, tumor localization and stage, and preoperative bowel preparation), intraoperative (e.g., surgical approach and technique, surgeon experience, and anastomosis height in rectal resection), and postoperative (e.g., necessity of blood transfusion) factors.

The identification of decisive risk factors is noteworthy because some can be influenced by preoperative bowel preparation. As early as 1994, Schardey et al. reported reduced AL rates with the oral application of antibiotics before surgery [13], which has been confirmed in various randomized clinical studies, such as the SELECT trial [14–16]. However, the antibiotic regimens used in these studies showed a wide range in both the type and number of antibiotics used and in the dosage regimen. Additionally, the exact protective mechanisms are not clearly understood. However, a shift in the gut microbiome to the disadvantage of collagenase-producing bacteria appears to play an important role [17].

This study aimed to assess the risk and protective factors of AL following colon and rectal cancer surgery at two certified colorectal cancer centers, including real patient care data regarding the period when oral antibiotics were introduced as a routine measure.

## Material and methods

### Study population

Two prospectively held databases were queried for all consecutive patients with colorectal cancer who underwent colorectal cancer surgery and received a primary anastomosis at Dresden-Friedrichstadt General Hospital (Center I) between 01.01.2012 and 31.12.2018 and at the University Hospital Leipzig (Center II) between 01.01.2017 and 31.12.2019. The data were supplemented by an extensive chart review and retrospectively analyzed. This study was approved by the Ethics Committee of the Medical University of Leipzig (number 437/24-ek). Patient consent was not required for this study, as confirmed by the local ethics committee.

### Inclusion and exclusion criteria

The inclusion criteria were age > 18 years, elective colorectal cancer surgery with bowel resection, and primary anastomosis (resections as planned secondary procedures after relief of the ileus by osteotomy were also included).

Exclusion criterion was: Multivisceral resection (planned or due to intraoperative findings).

### Preoperative variables and postoperative outcomes

The following variables were used to assess preoperative characteristics: age; sex; physical status according to the American Association of Anaesthesiologists (ASA) classification system (ASA grade I-II, ASA grade III–V); BMI (body mass index); obesity defined as BMI ≥ 30 kg/m^2^; previous abdominal operation; preoperative anemia (< 8.6 mmol/l for males and < 7.4 mmol/l for females); active smoking status; diabetes mellitus type II requiring treatment (oral anti-diabetic or insulin medication); cardiovascular risk factors (including coronary artery disease, arterial occlusive disease or previous stroke); anticoagulant therapy; ongoing immunosuppressive therapy; type of preoperative therapy (radiotherapy and/or chemotherapy); location of the colorectal carcinoma (right hemicolon, transverse colon, left hemicolon/sigmoid colon, upper (≥ 12–16 cm from the anal verge) vs middle (6– < 12 cm from the anal verge) vs lower rectum (< 6 cm from the anal verge); preoperative mechanical bowel preparation (MBP) with Klean-Prep® (Norgine, Germany) or MOVIPREP® (Norgine, Germany); preoperative oral antibiotic (OA) treatment: At center I, 375 mg sultamicillin and 500 mg metronidazol after MBP were administered at the evening before and at the morning of the operation (ciprofloxacin 500 mg instead of sultamicillin was given in case of intolerance), at center II, 4 g paromomycin and 1 g metronidazole were given after MBP the evening before the operation. In rare cases, if MBP was not possible due to stenosis, OA was administered without MBP. Intravenous administration of 3 g sultamicillin (center I) or 1 g ertapenem (center II) was administered 30 min prior to the incision and repeated according to each center’s policy.

Perioperative characteristics were reported with the following variables: Operation time; surgical technique (open surgery, minimally-invasive, converted); surgical procedure (extended) right hemicolectomy, (extended) left hemicolectomy/sigmoidectomy, rectum resection with partial mesorectal excision (PME), rectum resection with total mesorectal excision (TME), rectum resection with intersphincteric excision); surgeon’s experience (specialized colorectal surgeon, others); anastomotic technique (handsewn, stapled); intraoperative complications during the anastomosis (resection due to poor perfusion, the tension on the anastomosis, positive air leak test); protective stoma (ileostomy) that was created before or during the tumor operation; perioperative blood transfusions (during surgery and up to 10 days after the operation).

Postoperative outcomes were reported using the following variables: anastomotic leakage (AL) within 30 days after surgery, 30-day surgical site infections (SSI), complications according to the Clavien–Dindo classification [18] during in-hospital stay, staging according to the Union for International Cancer Control (UICC), and length of stay (LOS).

AL was defined according to an international consensus as defects of the intestinal wall at the anastomotic site leading to communication between the intra- and extraluminal compartments, requiring active therapeutic intervention without re-laparotomy (Grade B) or with re-laparotomy (Grade C) [19].

### Statistical analysis

Statistical analyses were performed using IBM SPSS Statistics for Windows version 29 (IBM Corp., Armonk, NY, USA). Continuous variables are expressed as the median and interquartile range (IQR) and compared using the Mann*–*Whitney *U-*test. Pearson’s chi square test was used to compare categorical variables. We performed a stepwise backward multivariate logistic regression model adjusting for age, ASA Grade, active smoking status, type II diabetes mellitus, cardiovascular risk factors, anticoagulation, ongoing immunosuppressive therapy, MBP, OA, surgical approach, surgeon’s experience, surgical procedure, type of anastomosis, intraoperative complications during anastomosis, and perioperative blood transfusions (additionally, protective stoma, previous therapy, and localization in the rectum for logistic regression in patients with rectal cancer). The results were reported as odds ratios (OR) and 95% confidence intervals (95% CI). All p-values were two-sided, and the significance level was set at *p* < 0.05.

The number of treatments needed was calculated as 1/absolute risk reduction. Absolute risk reduction was defined as the difference between the event rates in the group administered oral antibiotics preoperatively and that in the control group.

## Results

### Patients’ characteristics

We collected data from 717 consecutive patients, of whom 418 had colon cancer and 299 had rectal cancer. Males predominated in both cohorts (58.6% of patients with colon cancer and 68.6% of patients with rectal cancer). Patients with colon cancer were older (median age, 73 vs. 65 years). BMI and the number of obese patients were comparable between the two groups. Patients with colon cancer were more frequently categorized as ASA grade ≥ III and were more likely to have a cardiovascular risk profile, anticoagulant therapy, and anemia preoperatively.

Most patients with rectal cancer who received neoadjuvant therapy (mainly radiochemotherapy) were operated on by a colorectal surgeon, underwent a stapled anastomosis and a protective ileostomy. Surgical access remained open until 2016. OA (normally in combination with MBP, except in rare cases) has been performed for both entities since 2017 (center I) and 2019 (center II).

AL was present in 7.9% of the resected colon cancer cohort and in 10% of the rectal cancer cohort. Postoperative complications according to Clavien–Dindo were similarly distributed, whereas SSI was predominant in patients with colon cancer (9.3% vs. 2.7%) (Table 1).

**Table 1:** Characteristics of the study population

	Colon cancer *n* = 418	Rectum cancer *n* = 299
*Patient characteristics*		
Sex, *n* (%)		
Males	245 (58.6)	205 (68.6)
Females	173 (41.4)	94 (31.4)
Age (years), median (IQR)	73 (16)	65 (16)
BMI (kg/m^2^), median (IQR)	26.57 (5.31)	25.98 (6.28)
BMI ≥ 30 kg/m^2^, *n* (%)	89 (21.3)	71 (23.7)
ASA Grade, *n* (%)		
I–II	233 (55.9)	211 (70.6)
≥ III	184 (44.1)	88 (29.4)
*Missing values (n)*	*1*	
Anemia preoperatively, *n* (%)	227 (54.3)	127 (42.6)
*Missing values (n)*		*1*
Previous operation, *n* (%)		
Intestine	69 (16.6)	53 (17.7)
Other abdominal operation	160 (38.5)	64 (21.4)
*Missing value (n)*	*1*	
Active or previous		
smoking status, *n* (%)	88 (21.5)	69 (23.5)
*Missing value (n)*	*8*	*6*
Diabetes mellitus type II, *n* (%)	82 (19.6)	59 (19.8)
Cardiovascular disease, *n* (%)	74 (17.7)	27 (9)
*Missing value (n)*		*1*
Anticoagulation, *n* (%)	156 (37.3)	76 (25.4)
Immunosuppressants, *n* (%) *Missing value (n)*	10 (2.4) *2*	11 (3.7) *1*
Neoadjuvant therapy, *n* (%)		
Radio(chemo)therapy		175 (58.5)
Chemotherapy	9 (2.2)	14 (4.7)
MBP, *n* (%)	237 (56.7)	265 (89.2)
*Missing value (n)*		*1*
OA, *n* (%)	157 (37.7)	109 (36.6)
Type of oral antibiotics, *n* (%)		
Sultamicillin/Metronidazole	118 (28.4)	78 (26.2)
Ciprofloxacin/Metronidazole	9 (2.2)	6 (2)
Parmomomycin/Metronidazole	23 (5.5)	22 (7.4)
Others	7 (1.7)	3 (1)
*Missing value (n)*	*2*	*1*
*Tumor related variables*		
Tumor localization colon, *n* (%)		
Cecum	63 (15.1)	
Ascending colon	92 (22)	
Right flexure	29 (6.9)	
Transverse colon	34 (8.1)	
Left flexure	21 (5)	
Descending colon	19 (4.5)	
Sigmoid colon	160 (38.3)	
Tumor localization rectum, *n* (%)		
< 6 cm from anal verge		55 (18.4)
< 12 cm from anal verge		165 (55.2)
≥ 12 cm from anal verge		77 (25.8)
*Missing value (n)*		*2*
(y)UICC stage, *n* (%)		
0		26 (8.7)
I	91 (21.9)	92 (30.8)
II	142 (34.1)	55 (18.4)
III	107 (25.7)	67 (22.4)
IV	66 (15.9)	55 (18.4)
*Missing value (n)*	*12*	*4*
*Intraoperative variables*		
Operation time (min), median (IQR)	224 (92)	337 (136)
Approach, *n* (%)		
Open	288 (67)	199 (67)
Minimally-invasive	115 (27.5)	87 (29.3)
Minimally-invasive converted	10 (2.4)	11 (3.7)
*Missing value (n)*	*2*	*4*
Surgical procedure, *n* (%)		
(Extended) Right hemicolectomy/ Resection of Colon transversum	209 (50)	
(Extendend) Left hemicolectomy/ Sigmoidectomy	209 (50)	
Rectum resection with partial mesorectal excision		57 (19.1)
Rectum resection with total mesorectal excision		202 (67.6)
Rectum resection with intersphincteric excision		40 (13.4)
Surgeon’s experience, *n* (%)		
Specialized colorectal surgeon	166 (39.7)	242 (80.9)
General surgeons and surgical trainees	252 (60.3)	57 (19.1)
Patents with intraoperative complications during anastomosis, n (%)	15 (3.6)	29 (9.7)
Type of anastomosis, *n* (%)		
Hand-sewn	260 (63)	48 (16.2)
Stapler	146 (35.4)	235 (79.1)
*Missing value (n)*	*7*	*14*
Protective stoma, *n* (%)	19 (4.5)	250 (83.6)
*Postoperative outcomes*		
AL, *n* (%)	33 (7.9)	30 (10)
SSI, *n* (%)	39 (9.3)	8 (2.7)
Patients with blood transfusion perioperatively, n (%)	81 (19.4)	34 (11.4)
*Missing value (n)*	*1*	*2*
Postoperative complications according to Clavien–Dindo, *n* (%)		
I	24 (5.8)	14 (4.7)
II	64 (15.4)	41 (13.8)
IIIa	10 (2.4)	6 (2)
IIIb	49 (11.8)	28 (9.4)
IVa	7 (1.7)	7 (2.4)
IVb	2 (0.5)	0
V	7 (1.7)	4 (1.3)
*Missing value (n)*	*1*	
LOS (days), median (IQR)	12 (8)	12 (6)
*Missing value (n)*	*1*	*3*

*IQR* interquartile range, *BMI* body mass index, *ASA* American association of anaesthesiologists, *MBP* mechanical bowel preparation, *OA* oral antibiotics preoperatively, *UICC* union for international cancer control, *AL* anastomotic leakage, *SSI* surgical site infections, *LOS* length of stay

### Model quality of the logistic regression

Missing values were not included in the logistic regression analysis. All variables were tested for linearity, which was confirmed. Multicollinearity was not observed. The binomial logistic regression model was statistically significant (χ^2^(23) = 35.554, *p* = 0.046 for colon cancer surgery and χ^2^(29) = 48.104, *p* = 0.014 for rectal cancer surgery). The explanatory power of the model, as described by *Nagelkerke's* R^2^, was R^2^ = 0.207 for the colon and R^2^ = 0.335 for the rectum. The goodness of fit of the model, which was tested using Hosmer–Lemeshow, can be rated as high, χ^2^(8) = 9.967 *p* = 0.267 for the colon and χ^2^(8) = 3.627 *p* = 0.889 for the rectum.

### Risk and predictive factors for anastomosis leakage

#### Colon cancer resections

AL occurred in 33 (7.9%) patients who underwent surgery for colon cancer. Multivariate analysis showed that AL was more likely for (extended) left hemicolectomy/sigmoidectomy procedures as compared to (extended) right hemicolectomy (OR 3.771; 95% CI 1.17–12.154; *p* = 0.026) and for the perioperative administration of blood transfusions (OR 2.926; 95% CI 1.013–8.455; *p* = 0.047).

OA (usually in combination with MBP) before surgery was the only independent factor associated with a reduced AL (OR 0.199, 95% CI, 0.064–0.617; *p* = 0.005). The type of OA-regime regimen applied was not statistically significant (univariate analysis, *p* = 0.232) (Table 2).

**Table 2:** Multivariate analysis of risk factors for AL (colon cancer resections)

	AL *n* = 33	No AL *n* = 385	Multivariate analysis		
			OR	95%CI	*p*
Sex, *n* (%)					0.166
Males	23 (69.7)	222 (57.7)			
Females	10 (30.3)	63 (42.3)			
Age (years), median (IQR)	72 (15)	70 (15)			1.011
BMI ≥ 30 kg/m^2^	10 (30.3)	79 (20.5)			0.166
ASA Grade, *n* (%)					1.052
I–II	17 (51.5)	216 (56.3)			
≥ III	16 (48.5)	168 (43.8)			
Active or previous smoking status, *n* (%)					0.433
yes	7 (21.1)	81 (21.5)			
no	26 (78.8)	296 (78.5)			
Diabetes mellitus type II, *n* (%)					0.542
yes	8 (24.2)	74 (19.2)			
no	25 (75.8)	311 (80.8)			
Cardiovascular disease, *n* (%)					0.217
yes	10 (30.3)	86 (22.3)			
no	23 (69.7)	299 (77.7)			
Anticoagulation, *n* (%)					0.600
yes	14 (42.4)	142 (36.9)			
no	19 (57.6)	243 (63.1)			
Immunosuppression, *n* (%)					0.538
yes	1 (3.1)	9 (2.3)			
no	31 (96.9)	375 (97.7)			
MBP, *n* (%)					0.385
yes	23 (69.7)	214 (55.6)			
no	10 (30.3)	71 (18.4)			
OA, *n* (%)					
yes	7 (21.2)	150 (39.2)	0.199	0.064–0.617	**0.005**
no	26 (78.8)	233 (60.8)			
Approach, *n* (%)					0.996
Open surgery	24 (75)	264 (63)			
Minimally-invasive	7 (21.9)	108 (35.2)			
Minimally-invasive converted	1 (3.1)	9 (2.4)			
Surgical procedure, *n* (%)					
(Extended) Right hemicolectomy	11 (33.3)	198 (51.4)			
(Extendend) Left hemicolectomy/					
Sigmoidectomy	22 (66.7)	187 (48.6)	3.771	1.17–12.154	**0.026**
Surgeon’s experience, *n* (%)					0.237
Specialized colorectal surgeon	16 (48.5)	150 (39)			
General surgeons and surgical trainees	17 (51.5)	235 (61)			
Patients with intraoperative complications during anastomosis, *n* (%)	2 (6.1)	13 (3.4)			0.721
Type of anastomosis, *n* (%)					0.999
Hand-sewn	20 (62.5)	240 (63)			
Stapler	12 (37.5)	134 (35.2)			
Patients with blood transfusions perioperatively, *n* (%)	11 (66.7)	17 (18.4)	2.926	1.013–8.455	**0.047**

*AL* anastomotic leakage, *IQR* interquartile range, *BMI* body mass index, *ASA* American association of anesthesiologists, *MBP* mechanical bowel preparation, *OA* preoperative oral antibiotics

#### Rectal cancer resections

Thirty (10%) patients who underwent rectal cancer surgery had AL. A BMI ≥ 30 kg/m^2^ increased the probability of AL (OR 7.136; 95% CI 2.248–12.651; *p* < 0.001). OA before surgery was the only independent factor associated with a reduced AL rate in the multivariate analysis (OR 0.278, 95% CI, 0.078–0.985; *p* = 0.047). However, the OA type was not statistically significant (univariate analysis, *p* = 0.192) (Table 3).

**Table 3:** Multivariate analysis of risk factors for AL (rectal cancer resections)

	AL *n* = 30	No AL *n* = 269	Multivariate analysis		
			OR	95%CI	*p*
Sex, *n* (%)					0.113
Males	25 (83.3)	180 (66.9)			
Females	5 (16.7)	89 (33.1)			
Age (years), median (IQR)	63 (10)	64 (8)			0.534
BMI ≥ 30 kg/m^2^	13 (43.3)	58 (21.6)	7.136	2.248–12.651	** < 0.001**
ASA Grade, *n* (%)					0.261
I–II	23 (26.7)	188 (69.9)			
≥ III	7 (23.3)	81 (30.1)			
Active or previous smoking status, *n* (%)					0.511
yes	8 (26.7)	61 (23.2)			
no	22 (73.3)	202 (76.8)			
Diabetes mellitus type II, *n* (%)					0.057
yes	5 (16.7)	54 (20.1)			
no	25 (83.3)	214 (79.9)			
Cardiovascular disease, *n* (%)					0.372
yes	4 (13.3)	36 (13.4)			
no	26 (86.7)	23 (86.6)			
Anticoagulation, *n* (%)					0.529
yes	9 (30)	67 (24.9)			
no	21 (70)	202 (75.1)			
Immunosuppression, *n* (%)					0.694
yes	1 (3.3)	10 (3.7)			
no	29 (96.7)	258 (96.3)			
Previous therapy, *n* (%)					0.449
Chemotherapy	1 (3.3)	13 (4.8)			
Radio(chemo)therapy	20 (66.7)	155 (57.6)			
no	9 (30)	101 (37.5)			
Tumor localization, *n* (%)					0.564
< 6 cm from anal verge	4 (13.3)	51 (19)			
≥ 6 cm from anal verge	26 (86.7)	218 (81)			
MBP, *n* (%)					0.656
yes	25 (86.2)	240 (89.6)			
no	4 (13.8)	28 (10.4)			
OA, *n* (%)					
yes	6 (20)	103 (38.4)	0.278	0.078- 0.985	**0.047**
no	24 (80)	165 (61.6)			
Approach, *n* (%)					0.208
Open surgery	19 (67.9)	180 (66.9)			
Minimally-invasive	7 (25)	80 (29.7)			
Minimally-invasive converted	2 (7.1)	9 (3.3)			
Surgical procedure, *n* (%)					0.284
Rectum resection with partial mesorectal excision	4 (13.3)	53 (19.7)			
Rectum resection with total mesorectal excision	21 (83.3)	177 (65.8)			
Rectum resection with intersphincteric excision	1 (3.3)	39 (14.5)			
Surgeon’s experience, *n* (%)					0.416
Specialized colorectal surgeon	24 (80)	218 (81)			
General surgeons and surgical trainees	5 (20)	51 (19)			
Patients with complications during anastomosis, *n* (%)	5 (16.7)	24 (8.9)			0.185
Type of anastomosis, *n* (%)					0.555
Hand-sewn	5 (16.7)	43 (16.1)			
Stapler	24 (80)	211 (79)			
Protective stoma, *n* (%)					0.649
yes	27 (90)	224 (83.3)			
no	3 (10)	45 (16.7)			
Patients with blood transfusions perioperatively, *n* (%)	3 (10.3)	31 (11.6)			0.634

*AL* anastomotic leakage, *IQR* interquartile range, *BMI* body mass index, *ASA* American association of anesthesiologists, *MBP* mechanical bowel preparation, *OA* preoperative oral antibiotics

#### Number needed to treat

The calculated number of patients required for treat was 15.94. Therefore, 16 patients required treatment with oral antibiotics prior to colorectal cancer surgery to prevent AL.

## Discussion

We identified left-sided resection and perioperative administration of blood transfusions in elective colon cancer surgery and obesity in elective rectal cancer resection as risk factors for AL. The only protective factor in both groups was oral antibiotic bowel preparation (usually combined with mechanical bowel preparation), regardless of the regimen used. These results are based on a large prospectively documented patient cohort of over 700 patients with colorectal cancer and make an important contribution to the reflection of real-life conditions within the framework of a non-randomized, bi-center study.

Male sex [20–22] and, notably, younger age were found to be risk factors for colorectal AL in multivariate, national studies [23]. Smoking [24, 25], underlying disease affecting the vascular system or diabetes, as well as the presence of other severe diseases/comorbidities corresponding to ASA ≥ 3 [26], and the use of immunosuppressants [27] have also been shown to be potential risk factors. None of these risk factors was confirmed in our study. Smoking and diabetes were only present in a maximum of 20% of our study population, and immunosuppressant use affected < 4% of patients. This might explain why these variables were not identified as risk factors. However, consistent with a recent meta-analysis where obesity (BMI ≥ 30 kg/m^2^) increased AL in rectal resection with an OR of 1.57 in the Western population [28], we also identified obesity to be associated with a higher risk for AL in rectal resections (OR 7.136 (95% CI 2.248–12.651; *p* < 0.001). This is mainly due to the limited access during the surgical procedure, which might hamper the successful creation of an anastomosis in the narrow anatomical conditions of the small pelvis. Malnutrition is another important preoperative risk factor. However, in our cohort, none of the patients who were underweight (BMI < 18.5 kg/m^2^) had AL. However, it must be noted that only 10 patients were diagnosed as being underweight.

Bowel preparation plays a special role as a preoperative factor, owing to its potential as an intervention. The type of bowel preparation to be performed prior to colorectal surgery is an ongoing, intensive debate. OA usually in combination with MBP was shown in our current study to be a protective factor for AL in both, the colon (OR 0.199, 95% CI 0.064–0.617; *p* = 0.005) and the rectum (OR 0.278, 95% CI 0.078–0.985; *p* = 0.047) with a number needed to treat of 16 patients. However, MBP alone did not exert a protective effect. Our findings are consistent with the results of current meta-analyses, which have shown strong evidence for a reduced risk of AL after MBP/OA (OR 0.57, 95% CI 0.3–0.89, *p* = 0.003 compared to MBP alone [16]) [12, 29, 30]. The *American Society of Colon and Rectal Surgeons* strongly recommends combined bowel preparation (MBP/OA) in its 2019 updated guidelines [31] and the German S3-Guideline for PeriOperative Management for Gastrointestinal Tumors (POMGAT, 2023) followed [32]. In contrast, preoperative antibiotic bowel preparation is not yet part of day-to-day practice in surgical units [33, 34]. There is evidence that OA alone is sufficient; however, further randomized studies are required. Moreover, the pathway of interaction between the gut microbiome and AL remains unclear.

The guidelines do not provide recommendations regarding the type of oral antibiotic treatment. Existing studies are extremely heterogeneous regarding the type and regimens of antibiotics, ranging from a single dose of one antibiotic to several days of up to four antibiotics [15, 35]. Our study shows that the less complex regimens of two oral antibiotics (sultamicillin, in the case of an allergy, ciprofloxacin/metronidazole administered twice, or a single administration of paromomycin/metronidazole) at the two participating centers are equally effective. The investigation of different protocols in the same setting was a unique feature of our study. Further randomized studies are needed to compare different regimens and determine the least burdensome regimen for patients.

Regarding colon surgery, left-sided hemicolectomy/sigmoidectomy was found to be a risk factor compared to right-sided resection (OR 3.771; 95% CI 1.17–12.154; *p* = 0.026). This confirms the results of Krarup et al., who found an OR of 2.02 (95% CI 1.50–2.72; *p* = 0.01) for left hemicolectomy and an OR of 1.69 (95% CI 1.32–2.17; *p* = 0.01) for sigmoid colectomy in their nationwide Danish prospective cohort study [36]. By contrast, anastomosis in the lower third of the rectum was not a risk factor for AL in our study. This might be because a protective ileostomy is always placed in low rectal resections in accordance with hospital standards and the German S3-guideline for colorectal carcinoma.

Intraoperative factors such as surgeon experience, surgical approach, type of anastomosis, and reported complications during anastomosis were not identified as risk factors for AL in our patient cohort. Surgeon experience as an independent risk factor has been repeatedly discussed in the literature. A meta-analysis by Kelly et al. [37] found no differences in AL rates between trainees and specialist surgeons. A Cochrane Review by Archampong et al. [38] found that postoperative mortality and permanent stoma placement depended on the surgeon's caseload. Both centers are certified high-volume colorectal cancer centers, in which almost all colon and rectal operations are performed or supervised by specialized colorectal surgeons. Data regarding the superiority of one of the two anastomotic techniques (hand-sewn or stapled) are heterogeneous [39–42]. However, prospective randomized trials comparing techniques in the right colon are lacking. Intraoperative complications of anastomosis (e.g., a positive air leak test or renewed anastomosis placement due to poor perfusion) were not risk factors for AL in our study. Generally, the relationship between intraoperative complications during anastomosis and AL has rarely been investigated using multivariate analyses [19, 43]. If so, the definition was broad and included intraoperative bleeding and the administration of blood transfusions, which in our study represented a risk factor.

It is important to highlight that our study encompasses an 8-year period, with the administration of oral antibiotics predominantly occurring in the later years. This temporal bias should be taken into consideration. The most significant change was the transition from open surgical access to a predominantly laparoscopic/robotic approach. Nevertheless, it is essential to note that the type of surgical approach does not appear to exert a significant effect on the incidence of anastomotic leakage, as corroborated by findings from other studies in the literature [4, 44]. Additionally, new techniques, such as the use of indocyanine green for visualizing anastomotic blood flow, were explored, though their application was somewhat inconsistent. The core principles of the Enhanced Recovery Protocol—such as early mobilisation, timely removal of urinary catheters, prompt reintroduction of nutrition, and, when necessary, bowel stimulation—were already in practice at the beginning of the study period, but became more structured and comprehensive over time.

Our results showed that the administration of blood transfusions during and within 10 days after surgery was a risk factor for AL in colon cancer surgeries (OR, 2.926; 95% CI 1.013–8.455; *p* = 0.047). This finding is consistent with the retrospective analysis by Marinello et al. (OR 2.83, 95% CI 1.59–5.06, *p* < 0.0001) [45] and with Krarup et al. [34], who found an OR of 10.27 (95% CI 6.82–15.45; *p* < 0.001) for perioperative blood transfusions in their nationwide Danish trial. Patients with rectal cancer in our cohort generally required perioperative blood transfusion significantly less often, which was also potentially influenced by the fact that only 25% of the patients with rectal cancer vs. 37% of the patients with colon cancer received anticoagulation therapy and fewer patients presented with preoperative anemia (42.6% vs. 54.3%). This could explain why perioperative blood transfusion during rectal surgery was not a risk factor for AL in our study, in contrast to the findings reported by other studies [46].

The strength of our study is the real-world setting of patient care, which, owing to its consecutive nature, offers advantages over randomized trials. We can refer to well-documented data from over 700 patients over a period of eight years, and the inclusion of two colorectal surgery centers makes our study more stable against surgeon-related bias. However, this study has some limitations that need to be addressed. First, our investigation, although based on prospective databases, was a retrospective analysis of all inherent problems with this kind of study, despite the inclusion of a large number of patients. Second, the low insufficiency rate resulted in only a small number of events, which somewhat limited the validity of the multivariate analysis. In addition, over the long duration of the study—with the introduction of oral antibiotics in the final third—several modifications were made to the surgical procedure, most notably the shift to a predominantly minimally invasive approach and the strengthened implementation of the colorectal Enhanced Recovery Programme. A further limitation of the bi-centric design lies in the slight differences between centers with respect to the antibiotic regimen applied. While both centers followed the evidence-based principle of combining adequate aerobic and anaerobic oral antibiotics—which has been demonstrated to be the most effective strategy—the specific aerobic agents used differed.

## Conclusion

Our study confirms left-sided anastomosis and perioperative blood transfusion as well as obesity in elective colorectal cancer surgery as risk factors for AL. We identified preoperative oral antibiotic administration (usually with MBP) as a single protective factor against AL during bowel preparation in both entities. Both the less complex antibiotic regimens (one or two doses of two well-tolerated antibiotics) were equally effective. Bowel preparation for OA is an easy-to-implement procedure that should be the standard of care in elective bowel surgery.

## Supplementary Information

Below is the link to the electronic supplementary material.

Supplementary file1 (DOCX 17 KB)

## Data Availability

Data are available on reasonable request.
