# Correction to: Risk and protective factors for anastomotic insufficiency in elective colon and rectal cancer resections – a multivariate analysis with over 700 patients

**DOI:** 10.1007/s00423-026-04008-3

**Published:** 2026-03-10

**Authors:** Undine Gabriele Lange, Matthias Mehdorn, Gudula Keller, Anja Willing, Holger Nitzsche, Leif Christian Wagner, Ines Gockel, Boris Jansen-Winkeln, Soeren Torge Mees, Sigmar Stelzner

**Affiliations:** 1 Department of Visceral, Transplant, Thoracic and Vascular Surgery, University Hospital Leipzig, 04103 Leipzig, Germany; 2 Orthopedic Surgery, Caspar-David-Friedrich Str. 15, 01217 Dresden, Germany; 3 Department for General and Visceral Surgery, Dresden-Friedrichstadt General Hospital, 01067 Dresden, Germany; 4 Department for General, Visceral, Thoracic and Vascular Surgery, Federal Armed Forces Hospital Koblenz, 56072 Koblenz, Germany; 5 University Center for Gastrointestinal and Liver Disease, Clarunis, 4002 Basel, Switzerland; 6 Department for General, Visceral, Thoracic and Vascular Surgery, Hospital St. Georg Leipzig, 04129 Leipzig, Germany

**Correction to: Langenbeck’s Archives of Surgery (2026) 411:54**

10.1007/s00423-025-03953-9

The original version of this article unfortunately contained an error. The author’s name ‘Gudula Keller’ was incorrectly written as ‘Gudrun Keller’.

The original article has been corrected.

