# Supplementary material for: Risk and protective factors for anastomotic insufficiency in elective colon and rectal cancer resections – a multivariate analysis with over 700 patients
Source: Langenbecks Arch Surg. 2026 Jan 9;411(1):54. doi: 10.1007/s00423-025-03953-9 (PMC12804435; doi:10.1007/s00423-025-03953-9)
Supplement: Supplementary file 1 — Supplementary file1 (DOCX 17 KB) [file 423_2025_3953_MOESM1_ESM.docx]

**Supplemental data Table 1: Specification of pre-, intra- and postoperative variables**

| Variable | Specification |
| --- | --- |
| age |  |
| sex | male or female |
| physical status according to ASA classification system | ASA grade I-II or ASA grade III–V |
| body mass index |  |
| obesity | defined as BMI ≥ 30 kg/m^2^ |
| previous abdominal operation |  |
| preoperative anemia | < 8.6 mmol/l for males and  < 7.4 mmol/l for females |
| active smoking status |  |
| diabetes mellitus type II | requiring treatment (oral anti-diabetic or insulin medication) |
| Atherosclerotic vascular disease | including coronary artery disease, arterial occlusive disease or previous stroke |
| anticoagulant therapy |  |
| ongoing immunosuppressive therapy |  |
| type of preoperative therapy | radiotherapy and/or chemotherapy |
| location of the colorectal carcinoma | right hemicolon, transverse colon, left hemicolon/sigmoid colon, upper (≤ 12–16 cm from the anal verge) vs middle (> 6– < 12 cm from the anal verge) vs lower rectum (< 6 cm from the anal verge) |
| preoperative mechanical bowel preparation | Klean-Prep® (Norgine, Germany) or MOVIPREP® (Norgine, Germany) |
| preoperative oral antibiotic treatment | center I: 375 mg sultamicillin and 500 mg metronidazol after MBP were administered at the evening before and at the morning of the operation (ciprofloxacin 500 mg instead of sultamicillin was given in case of intolerance)  center II: 4 g paromomycin and 1 g metronidazole were given after MBP the evening before the operation.  In rare cases, if MBP was not possible due to stenosis, OA was administered without MBP |
| surgical technique | open surgery, minimally-invasive, converted |
| surgical procedure | (extended) right hemicolectomy, (extended) left hemicolectomy/sigmoidectomy, rectum resection with partial mesorectal excision (PME), rectum resection with total mesorectal excision (TME), rectum resection with intersphincteric excision |
| surgeon’s experience | specialized colorectal surgeon, others |
| anastomotic technique | Handsewn or stapled |
| intraoperative complications during the anastomosis | resection due to poor perfusion, the tension on the anastomosis, positive air leak test |
| protective stoma (ileostomy) | that was created before or during the tumor operation |
| perioperative blood transfusions | during surgery and up to 10 days after the operation |
| anastomotic leakage | within 30 days after surgery |
| surgical side infections | within 30 days after surgery |
| complications according to the Clavien–Dindo classification |  |
| tumor stage according to the Union for International Cancer Control (UICC) |  |
| length of stay |  |

ASA = American Association of Anesthesiologists
